# Assessment of Nutritional Status and Dietary Pattern of a Rural Adult Population in Dry Zone, Sri Lanka

**DOI:** 10.3390/ijerph17010150

**Published:** 2019-12-24

**Authors:** Hansani Madushika Abeywickrama, K. M. Swarna Wimalasiri, Yu Koyama, Mieko Uchiyama, Utako Shimizu, Rohana Chandrajith, Nishantha Nanayakkara

**Affiliations:** 1 Graduate School of Health Sciences, School of Health Sciences, Faculty of Medicine, Niigata University, 2-746, Asahimachi, Niigata 951-8518, Japan; 2 Department of Food Science and Technology, Faculty of Agriculture, University of Peradeniya, Peradeniya 20400, Sri Lanka; 3 Department of Geology, Faculty of Science, University of Peradeniya, Peradeniya 20400, Sri Lanka; 4 Teaching Hospital, Kandy 20000, Sri Lanka

**Keywords:** diet, nutritional status, anthropometry, adults, dry zone of Sri Lanka, Girandurukotte

## Abstract

The objective of this work was to describe average dietary intake, physical activity (PA) and nutritional status of the adult population of Girandurukotte, Sri Lanka. A cross-sectional survey, including one 24-h dietary recall, international physical activity questionnaire and anthropometric measurements was conducted in a representative sample of 120 adults. Mean (SD) for body mass index (BMI), waist circumference (WC), waist to hip ratio (WHR) and waist to height ratio (WHtR) were 23.06(4.20) kg/m^2^, 85.6(9.5) cm, 0.95(0.05) and 0.55(0.07), respectively. Significant differences were observed in height, body fat %, body muscle %, hip circumference, WHR, WHtR, fat mass index and hand grip strength between men and women (*p* < 0.05). Among the study group, 35.8% were overweight, 13.3% were obese and 11.7% were underweight. Central obesity was observed in 59.2%, 97.5% and 74.2% of adults by WC, WHR and WHtR, respectively. Mean (SD) dietary diversity score and dietary diversity score with portions were 4.77(1.28) and 4.09(1.32), respectively. Mean daily intake of protein, fruits, vegetables and dairy were well below the national recommendations. Despite the higher PA level, nearly half the population was overweight and obese and the majority was centrally obese. None of the dietary diversity scores met the optimal levels, suggesting poor quality and quantity of the diet.

## 1. Introduction

Diet, nutritional status and physical activity are recognized as major determinants of health and particularly of non-communicable diseases (NCDs). It has been widely recognized that the global NCD epidemic is associated with recent life style changes, especially dietary and nutritional factors. Sri Lanka is a developing country undergoing substantial changes in its demographic and socio-economic environments, which imposed significant challenges on health and nutrition. Nutrition transition of the country is characterized by co-existence of over nutrition, under nutrition and micronutrient deficiencies [1] and higher prevalence of NCDs [2,3]. Girandurukotte is located in the Uva province, dry zone of the country where the majority of the population is involved in farming as the main occupation. Recently, this area has been identified as a hotspot of chronic kidney disease (CKD) of uncertain etiology (CKDu), which is an emerging form of kidney disease that is not attributed to any conventional risk factors such as hypertension, diabetes and glomerulonephritis [4]. Since malnutrition, selenium deficiency and foods as entry routes of nephrotoxins have been pointed out as possible contributors to the onset of the disease [5,6], surveys on the diet and nutrition of CKDu-affected populations are essential in order to identify possible nutritional and dietary factors that contribute to the onset and progression of the disease. In view of this situation, the aim of this study was to describe the dietary pattern and nutritional status and underlying demographic correlates among general adult population in Girandurukotte, Sri Lanka, with respect to the national and international recommendations.

## 2. Materials and Methods

### 2.1. Study Design and Participant Recruitment

A population-based cross-sectional survey was carried out in the Girandurukotte area located in Uva province, Sri Lanka. The survey was conducted from October to December 2018 on a randomly selected sample of 120 individuals over 18 years of age. Sample size was calculated using a sample size calculator for estimating a single proportion formula [7], assuming prevalence of obesity of 6.7% [8], 95% confidence level, 5% desired precision and 15% non-response rate to give 120. An updated electoral register obtained from the Grama Niladhari was used as the sampling reference. Individuals with psychiatric/cognitive disorders or language barriers, individuals who were extremely debilitated or participated in the pre-test and pregnant or lactating mothers were excluded from data collection.

Ethical clearance was obtained from the ethical review committees of the School of Health Sciences, Niigata University, Japan and the Faculty of Allied Health Sciences, University of Peradeniya, Sri Lanka (AHS/ERC/2018/021). Permission to conduct the study was obtained from relevant authorities in the Grama Niladhari division and Provincial government. An information sheet was provided prior to the invitation to participate in the survey and written consent was obtained from all participants.

### 2.2. Data Collection and Tools

Data collected were comprised of demographic and socio-economic, dietary, physical activity (PA) and anthropometric measurements. Door-to-door visits to randomly selected individuals were carried out and those who satisfied the inclusion criteria and willing to participate in the survey were recruited for the study. Questionnaires were pre-tested using 20 individuals. 

#### 2.2.1. Socio-Demographic Data

The socio-demographic variables collected were sex, age, education level, marital status, occupation, number of household members and level of income. In addition, the presence of NCDs and health-related habits such as alcohol consumption, cigarette smoking and betel chewing were also assessed.

#### 2.2.2. Dietary Data

A single 24-h dietary recall was used to assess dietary diversity and intake as it considered as the best reference period to assess dietary diversity [9]. The recalls were collected on random days by trained personnel to minimize day-to-day variation. In addition, a different date was selected for the interview if the previous 24 h period of an individual is atypical due to special occasion or illness. All foods and beverages consumed on the day before the interview were obtained in direct chronological order. Food intake was obtained using standard household measures, such as measuring cups, coconut shell spoons, tablespoons, teaspoons, glasses and teacups. Energy and macronutrient intake was analyzed using modified Nutrisurvey 2007 (EBISpro, Germany) nutrient analysis software. 

#### 2.2.3. Physical Activity Data

The long form of the international physical activity questionnaire (IPAQ) was translated into native language and the pre-tested questionnaire was used in data collection. The questionnaire covered four domains of PA: work-related, domestic and yard, transportation-related and leisure time. In addition, the IPAQ included questions about the time spent sitting. For each domain, the number of days over the last 7 days the participant spent more than 10 min walking, moderate and vigorous physical activity and duration per day were collected. All IPAQ data were cleansed and processed referring to the IPAQ scoring protocol [10]. Continuous measurements were expressed as metabolic equivalent of task minutes/week and individuals were classified as having ‘high’, ‘moderate’ or ‘low’ PA, based on their categorical score.

#### 2.2.4. Anthropometric Measurements

Measurements including height, weight, mid upper arm circumference (MUAC), waist circumference (WC), hip circumference (HC), body composition (% body fat and % muscle), blood pressure (BP) and handgrip strength (HGS) were obtained. Height was measured to the nearest 0.1 cm with an upright portable stadiometer (Seca 213; seca Deutschland, Hamburg, Germany) while the participant was in a standing position without footwear, looking straight ahead at the Frankfort plane. Weight and body composition measurements were obtained using a body composition monitor (Model HBF-220; Omron Healthcare Co. Ltd., Kyoto, Japan) by asking participants with minimal clothing and dry feet to step on the monitor after entering the participant’s gender, age and height into the machine. Guest mode was used throughout the period of study and values automatically displayed on the monitor were recorded for each participant. The MUAC, WC and HC were determined using a non-stretchable measuring tape (Seca 201) to the nearest 0.1 cm according to standard guidelines. WC was measured at the approximate midpoint between the lower margin of the last palpable rib and the top of the iliac crest. HC was measured at the widest circumference of the buttocks [11]. MUAC was measured at the midpoint between the acromion process and olecranon process when the forearm is hanging loose at the side [12]. BP was measured using a digital BP monitor (Model MP-126, Berlin, Germany). A systolic BP (SBP) of ≥ 140 mmHg and/or a diastolic BP (DBP) of ≥ 90 mmHg were considered as the cut-off levels for the presence of hypertension [13]. HGS was measured with a ZAZ electronic hand dynamometer (Zakka-town, Kumagaya, Japan) with participants seated in a chair with their hips flexed at 90° and feet resting on the floor without arm support. The shoulder of the rest arm was abducted and neutrally rotated, elbow was flexed to 90° and the forearm was in a neutral position. Following a demonstration, the dynamometer was placed vertically in line with the forearm and participant was asked to squeeze the handle with as much force as possible for 3 s [14]. Three repeated measurements were recorded for both hands with a rest period of at least 15 s between trials and the maximum value of the 3 trials was used in the analysis. 

### 2.3. Nutritional Status Indicators

Body mass index (BMI) was calculated as weight in kilograms divided by height in meters squared (kg/m^2^). Then, individuals were categorized based on the BMI cut-off values recommended for Asian populations by WHO expert consultation [15] as underweight (<18.5 kg/m^2^), normal (18.5–22.9 kg/m^2^), overweight (23–27.5 kg/m^2^) and obese (>27.5 kg/m^2^). Central obesity (CO) was defined according to WC, waist to hip ratio (WHR = WC/HC) and waist to height ratio (WHtR = WC/height). Men with a WC ≥ 90 cm and women with a WC ≥ 80 cm were identified as having CO, in accordance with the cut-off values for South Asians [16]. CO was also determined as a WHR ≥ 0.9 in men and ≥ 0.85 in women, according to the WHO criteria [11] and a WHtR of > 0.5 [17]. Individuals with a normal BMI but with CO were classed as having normal weight CO (NWCO). Fat mass index (FMI = fat mass/height^2^) was calculated as a measure of relative fat content.

### 2.4. Dietary Diversity Indicators

Dietary diversity of the participants was assessed by dietary diversity score (DDS), and DDS with portions (DDSP) using the 24-h dietary recall. These scores have been recognized as simple and useful indicators for a non-quantitative assessment of macro and micronutrient adequacy of the diet [18,19,20]. DDS was defined as the total number of different food groups consumed over a 24-h period, irrespective of the amount. Food groups considered were cereals, tubers and starchy vegetables, root and fruit vegetables, green leafy vegetables, fruits, pulses and legumes, meat and meat products, eggs, fish and seafood and milk and milk products. Therefore, the maximum score was 10, giving 1 point for each food group consumed by participant within the 24-h period. The selection of food groups was based on local and international food grouping methods adapted for cultural context [21,22,23]. However, food groups, such as oils and fats, sugar and sweets and spices, condiments and beverages, which are included as food groups proposed by Food and Nutrition Technical Assistance [21,22], were excluded from the current study since their consumption is much more common among the Sri Lankan population. DDSP was calculated applying a minimum consumption of one portion of each food group, as described above [24]. Hence, the maximum score was 10. DDS was identifies as a useful proxy indicator of nutrient adequacy of Sri Lankans [24,25]. Portion sizes for DDSP were determined in reference to the Food Based Dietary guidelines for Sri Lankans [23] and Jayawardene et al. [26].

### 2.5. Statistical Analyses

All statistical analyses were performed using SPSS 23.0 (SPSS, Inc., Chicago, IL, USA). Data were presented as frequencies (*n*) and proportions (%) for categorical variables, and mean values and standard deviations (SD) for continuous variables, unless otherwise specified. Significant differences in proportions between groups were calculated by the chi-square test. The *t* test for independent samples was used to compare means. Differences were regarded as significant at *p* < 0.05. A probability level of *p* < 0.05 was accepted as significant for all statistical tests. 

## 3. Results

The study population consisted of 68% (82 of 120) women and all the participants were Buddhists in religion. The socio-demographic and health-related characteristics of the study population are listed in Table 1. Among the betel chewers, 61.7% were men and all who drank alcohol and smoked cigarettes were men.

Mean values (SD) for anthropometric measurements and the indicators, BP and HGS are listed in Table 2. Significant differences were observed in height, % body fat, % body muscle, HC, WHR, WHtR, FMI and HGS between men and women. Among these parameters, % body fat, WC, WHtR and FMI were significantly higher in women, compared to men (*p* < 0.05). Mean SBP and DBP were higher than 140 and 90 mm/Hg, respectively, in both men and women, and based on these cut-off values, 58.3% of the study group were hypertensive. Among those who had not been previously diagnosed with hypertension, 52% had a SBP over 140 mm/Hg and/or a DBP over 90 mm/Hg.

Overall, 49% of the study population had a BMI higher than normal and 12% were underweight. CO by WC and WHtR was significantly higher in women compared to men (*p* < 0.05). Of the 39.2% with a normal BMI, 36.2%, 93.6% and 61.7% had CO by WC, WHR and WHtR, respectively. A significant difference was observed in the PA level between gender, while more than half (55%) of the population had a high PA level (Table 3). Average total sitting time per day was 140 (104) min.

**Figure 1:**
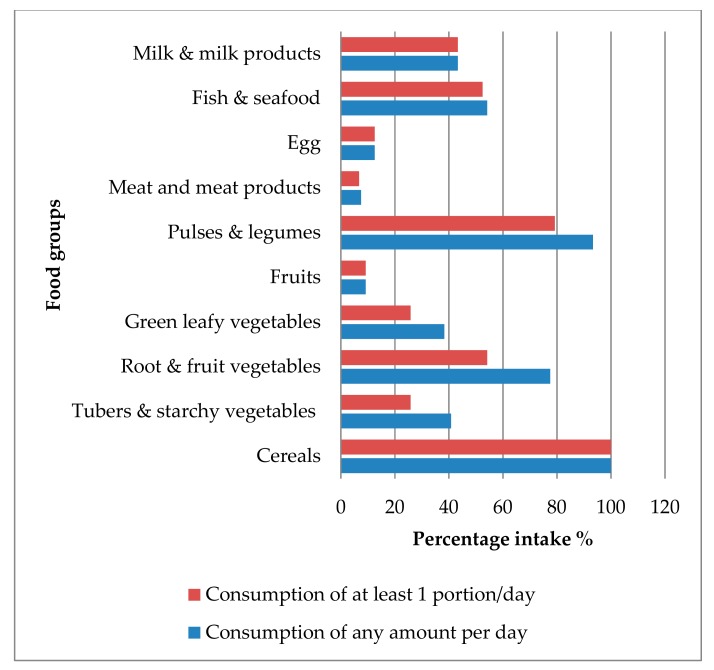
Consumption of different food groups during the previous 24-h period.

Dietary diversity and nutrient intake were assessed using 24-h dietary recalls and 53 (44.17%) of recalls represented the intake on weekend. The mean (SD) DDS of the study population was 4.77 (1.28) with a range of 2-8. DDSP was calculated applying consumption of at least one portion of each food group and resulted in a mean (SD) value of 4.09 (1.32) with a range of 1–7. Figure 1 shows the consumption of different food groups considering the consumption of any amount and at least one portion per day. All the study participants had consumed cereals and the majority had some kind of pulses or legumes (93.3%). Among animal foods, fish consumption (54.2%) was higher than eggs (12.5%) and meat and meat products (7.5%). Fruit consumption was less than 10%, while less than half of the study population had consumed tubers and starchy vegetables, green leafy vegetables or milk and milk products. None of the participants consumed nuts. The percentage of consumption of at least one portion per day of tubers and starchy vegetables (*p* = 0.02), root and fruit vegetables (*p* < 0.001), green leafy vegetables (*p* = 0.053) and pulses and legumes (*p* = 0.002) were significantly decreased, compared to the consumption of any amount per day. 

The mean intake of fruit and vegetables was extremely low, while the mean consumption of diary and plant and animal protein was below the national dietary recommendations for Sri Lankans. Only the mean intake of starch was within recommendations; however, 42.5% of the study population consumed cereals and equivalents in excess of the national recommendations. Consumption of plant and animal protein was higher than the recommended servings in 13.3% of the study population. Mean consumption of sugar exceeded the national recommendation and only 13.3% of the study population had not used added sugar in the previous 24-h period (Table 4). There was no significant difference between the mean intake of men and women for all the food groups considered below. Mean (SD) water intake was 6.46 (3.09) glasses per day with the range of 2–20. There was a statistically significant difference between mean water intake among men (7.39) and women (6.02).

Table 5 lists the daily energy and macronutrients (carbohydrate, protein and fat) intake of the study sample. There were no significant differences between these intakes among men and women, except for carbohydrate. In general, the energy intake was low among both genders. The mean macronutrient composition as a percentage of total energy from carbohydrate, protein and fat were 72.18%, 10.62% and 17.2%, respectively. Percentage contribution of fat and carbohydrates to daily energy consumption were significantly higher among females compared to males. Proportions of population within the acceptable recommended range for percentage energy from carbohydrate, protein and fat were 17.5%, 60% and 47.5%, respectively.

## 4. Discussion

Although a number of studies have been conducted on different aspects of CKDu, to the best of our knowledge, this is the first attempt to report the dietary intake and nutritional status of a CKDu-prevalent community in Sri Lanka. However, the aim of this study was to describe the general dietary pattern and nutritional status of this area, as a reference to future studies. The majority of the participants in this study had three or more family members (89.2%), and their educational status was poor. The income of 97% of the participants was above the poverty line for the Badulla district (SLR 4656) [27]. On average, the men were taller, heavier and had a higher % body muscle and HGS than the women. Conversely, % body fat and FMI were significantly higher in women than in men. There were no comparative data for adults over 18 years of age in Sri Lanka for body composition or HGS. However, our findings are similar to reported % body fat levels among middle-aged (34.7% [4.3%]) and elderly (36.7% [4.8%]) women, but higher than that of middle-aged (24.5% [4.6%]) and elderly (24.4% [5.5%]) men in Sri Lanka [28]. Our current findings are higher than the average % body fat and FMI reported for Chinese populations [29,30,31]. HGS is a simple non-invasive measure of physical strength and nutritional status [32]. The HGSs in the current study for both hands and both genders were weaker than those reported in Australia [33], Brazil [34], America [35], Europe [36,37], Iran [38] and Hong Kong [39], but higher than the values reported in Malaysia [40]. Our findings are in accordance with previous findings in that the HGS was lower in women than men [33,34,36,39,41,42,43]; this is most likely due to the significantly higher percentage of muscle mass observed in men, which is a major determinant of strength.

The mean (SD) BMI of the total study population was 23.06 (4.2) kg/m^2^, which is similar to the average BMI reported by Ranasinghe et al. (23.8 [4.2] kg/m^2^) [28], but higher than the mean BMI observed in a national survey (21.8 [4.2] kg/m^2^) [44] and in the Kaluthara district (22.9 [0.28] kg/m^2^) for adults [45]. The mean BMI for men in the present study is similar to the average BMI for men reported using national (21.1 [3.7] kg/m^2^) [8], provincial (Central Province, 22.7 [4.2] kg/m^2^ [46]; four provinces, 21.5 [3.7] kg/m^2^ [47]) and district (Colombo district, 22.8 [4.3] kg/m^2^ [48]; Kaluthara district, 21.9 [0.46] kg/m^2^ [45]) populations. Rathnayake et al. [49] reported a higher average BMI for adult women than in our present study; conversely, the average BMI for women reported by Katulanda et al. [8] (22.7 [4.5] kg/m^2^) was lower than the current study. However, an average BMI for women similar to the present study has also been reported [45,47,48]. The mean BMI of women was higher than that of the men which is similar to previous studies [44,45,47]. The mean WC of women (85.96 [9.53] cm) was higher than the Asian cut-off and previously reported values by Katulanda et al. (76.8 [12.2] cm) [8], De Silva et al. (79.4 [1.27] cm) [45] and Rathnayake et al. (78.51 [9.6] cm) [49]. The average WC for men (84.79 [9.38] cm) was less than the Asian cut-off, but higher than previously reported values. The average WHR and WHtR values for both men and women in the current study were higher compared to previous findings (WHR: men, 0.89 [0.07] cm versus women, 0.85 [0.08] cm [8]; WHtR: men, 0.48 [0.07] versus women, 0.5 [0.08] [50]). In accordance with Katulanda et al. [8] and Jayawardena et al. [50], we also found significant differences in WHR and WHtR between genders (*p* < 0.05).

Comparing the prevalence of BMI categories and CO can be a challenging task due to differences in the definitions used by authors. Four categories of adults were identified in this study, based on general obesity using the BMI cut-off values for Asians; hence, studies that used the same definitions were selected for comparison. Nearly 37% of men in the current study were overweight, which is higher than previous findings of 27.4%, 31.8% and 22.6% by De Silva et al. [45], Jayawardene et al. [46] and Katulanda et al. [8], respectively. Conversely, the proportion of overweight women in the current study was less than the findings of De Silva et al. (38.7%) [45], but higher than the findings of Jayawardene et al. (20.2%) [51], Katulanda et al. (28%) [8] and Jayatissa et al. (28.7%) [52]. The prevalence of obesity was 7.9% and 15.9% among men and women, respectively, in the current study. According to a national survey in 2010, 7.2% of men and 11.3% of women were obese [8]. Another study [51] reported a 20.1% and 35.9% prevalence of obesity among men and women, respectively, which is much higher than our current findings. The prevalence of obesity among men in the Central province (12.3%) [46] and Kaluthara district (9.2%) [45] was also higher than our findings. However, Jayatissa et al. [52] reported an obesity prevalence (15.2%) among women similar to the current study.

Individuals with a normal BMI may have CO, and therefore, use of BMI alone results in an underestimation of the at-risk population. Hence, proxy measures of abdominal fat, such as WC, WHR and WHtR, were used in this study to assess the central fat distribution. The assessment of CO by WC is the most commonly used criterion in many studies in Sri Lanka, and the 31.6% and 72% prevalence of CO by WC among men and women, respectively, in the current study were much higher than previous findings [45,46,48,51,53]. For instance, Katulanda et al. [8] reported a 16.5% and 36.3% prevalence of abdominal obesity among men and women, respectively. Prevalence of CO by WC was significantly higher among women than men in the present study, similar to previous studies [45,48]. Nearly half of our study population was overweight or obese (>23 kg/m^2^); however, the prevalence of CO was much higher by all indicators. CO is a component of the metabolic syndrome and recognized as a better predictor of cardiovascular disease risk than general obesity. The prevalence of CO among individuals with a normal BMI ranged from 14.2%–36.7% by all three criteria. A study in South Africa reported an NWCO prevalence of 26.9%–36.9% using WC, WHR and WHtR [54]. Studies among Thai health workers and Chinese adults also report the prevalence of NWCO at 15.4% [55] and 13.9% [56], respectively. The higher prevalence of NWCO suggests the need to consider other anthropometric indices, in addition to BMI, in the assessment of nutritional status and identification of at-risk individuals.

A national survey reported an average (SD) SBP and DBP of 128.9 (19.4) mmHg and 75.2 (11.6) mmHg, respectively, for men and 125.9 (19.9) mmHg and 75.4 (11.1) mmHg, respectively, for women [57], which are higher than the mean BP values in our study. The prevalence of undiagnosed hypertension in our study population was 40.8%, which is higher than that of the urban Sri Lankan population (31.8%) [58]. Overall, the prevalence of hypertension was also higher compared to previous findings [58,59]. Only 16.7% of our study group were physically inactive, which is much less compared to the 38% prevalence of low PA among government officials in the Colombo district [60]. Interestingly, the prevalence of low PA was higher and vigorous PA was lower in our study population than in a national survey (low PA, 11.1%; vigorous PA, 60%) [44].

Optimal nutrition and healthy dietary patterns are essential to ensure positive health outcomes. The food-based dietary guidelines in Sri Lanka have promoted the diversification of the daily diet in order to achieve recommended intakes of macro- and micro-nutrients for a healthy life. Our findings are in accordance with the mean DDS (4.4) among a group of rural elderly people. However, the above study only considered six food groups when calculating DDS [25]. Jayawardene et al. [24] reported a much higher mean DDS (men, 6.23; women, 6.5) among adult Sri Lankans, but a lower mean DDSP (men, 3.26; women, 3.17). A reduction in the percentage consumption of food groups when portions are taken into consideration indicates that the amount of consumption is low for many food groups, similar to previous findings [24,25]. In our attempt to identify food consumption according to servings per day, a low mean daily intake of all the food groups considered was observed, except for starchy foods and added sugar. Similar findings have been reported among a group of institutionalized elderly people [61]. Jayawardene et al. [26] reported mean daily intakes of fruits, vegetables and dairy among Sri Lankan adults that were well below the national recommendations, but comparable to our findings.

Amongst the food groups, the mean daily intake of fruits and vegetables least frequently met the national recommendations, which may be affected by purchasing ability, seasonal availability or the nutritional knowledge of the population. The study group consumed a large number of starch servings, and 42.5% consumed well above the upper cut-off of the national recommendations. This can be mostly due to the consumption of a monotonous diet which combines rice or rice product with a starchy vegetable such as potato, breadfruit, jackfruit, manioc and/or pulses such as lentil curry. Although pulses are grouped in the protein category, they invariably mask significant amounts of carbohydrate. Moreover, the majority (87%) of the study population consumed added sugar in excess of the national recommendations. This predominately carbohydrate diet may be a contributing factor to the high prevalence of general obesity and CO amongst the study population. Siriwardhana et al. [62] conducted a dietary investigation in the CKDu-prevalent Madawachchiya area of Sri Lanka. They reported that animal sources (meat, fish and eggs) most commonly accompany meals, followed by fruit vegetables, pulses and starchy vegetables. Consumption of 6–8 200mL glasses of water per day has been recommended for Sri Lankan adults [29]. The average intake of water was within the acceptable range; however, 47% of the study participants reported low consumption of water than recommended. Studies assessing the prospective risk of developing CKDu and water consumption are needed for this population.

Jayawardena et al. [63] studied the energy and macronutrient intake among Sri Lankan adults using a nationally representative sample and reported daily energy consumption among men (1913 + 567 kcal) and women (1514 + 458 kcal), which are much higher than the results of current study. In general, the average energy intake of study participants was below the requirements [64], contrary to the higher proportions of overweight and central obesity observed. This can be mainly due to the tendency of individuals to underreport the quantity of dietary intake, especially those who are overweight or obese. Moreover, this can be caused by some measurement bias in the study method, especially the use of a single 24 h dietary recall. The total daily intakes of carbohydrate, protein and fat were also lower in the study sample compared to corresponding values reported for Sri Lankan adults (304.4 g, 44.6 g and 35 g, respectively) [63]. WHO recommends proportion contribution to daily energy consumption of 55–75%, 15–30% and 10–15% from carbohydrates, fat and protein, respectively [65]. According to the previous findings, the proportion contribution of carbohydrate, protein and fat towards daily energy consumption of Sri Lankan adults was 71.2%, 10.8% and 18.9%, respectively, which is in line with the findings of our study [63]. However, 42% of the study group reported a total carbohydrate intake above 75% of total energy intake and 34.2% had total protein intake below 10% of total energy intake. More importantly, proportionate contribution of fat to total energy intake was less than 15% among 46.7% of the study population, which is the minimum requirement to ensure the demands for essential fatty acids.

A major strength of our study was the representation of all age, education and income groups of the community. The use of standard procedures and various anthropometric measures provide further credence to the study findings. Sample size was small, but thought to be representative of the study population with a low population density. Furthermore, representation of the male gender was relatively low in the study group, mostly due to their absence from home during the day time. Although a 24-h dietary recall may not be the best tool to accurately determine an individual’s habitual diet, a 24-h dietary recall provides estimations of population means. We conducted dietary recalls on both weekends and weekdays, in order to get a better evaluation of the usual dietary pattern of the population. Indeed, recall bias and over- and under-estimation are associated with the dietary recall method; however, we used visual aids and common household measures in order to minimize such errors. Although the findings of this study might not be representative of all CKDu-prevalent communities, which may limit generalization of the results, our study, nevertheless, provides a snapshot of the general nutritional status and dietary pattern of adults in this setting and assists health and nutritional policy-makers in developing and implementing specific programs to promote healthy dietary habits among the rural population in Sri Lanka.

## 5. Conclusions

We found a high prevalence of CO and hypertension among adults in this setting. These findings could be due to the lifestyle or genetic characteristics of this study group, which make them vulnerable to increased adiposity. None of the DDSs of the study population met the optimal levels, suggesting that both quality and quantity of the diets were poor. We observed an alarmingly low consumption of certain food groups compared to national recommendations, especially fruits and vegetables. Consumption of predominately high levels of carbohydrates and added sugar and low levels of fruits and vegetables may have detrimental effects on health and indicate prevalence of undiagnosed NCDs. These results could be used in this population to ensure healthy eating patterns and as a basis for future studies.

## Figures and Tables

**Table 1:** Socio-economic and health-related characteristics of the study population.

Variable	Frequency	Percentage (%)
Age		
18–29	17	14.2
30–39	38	31.7
40–49	19	15.8
50–59	15	12.5
60–70	22	18.3
>70	9	7.5
Education level		
No proper education	13	10.8
Primary	78	65
Secondary	25	20.8
Tertiary	4	3.3
Marital status		
Married	108	90
Divorced	1	0.8
Widowed	6	5
Unmarried	5	4.2
Number of family members		
1–2	13	10.8
3–5	66	55
>5	41	34.2
Monthly income (SLR)		
<5000	8	6.7
5001–10,000	8	6.7
10,001–20,000	28	23.3
20,001–30,000	53	44.2
30,001–50,000	16	13.3
>50,000	7	5.8
On treatment for NCDs		
Hypertension	26	21.7
Diabetes mellitus	13	10.8
Hypercholesterolemia	15	12.5
Coronary heart diseases	4	3.3
Chronic respiratory diseases	9	7.5
Health-related habits		
Smoking cigarettes	11	9.2
Betel chewing	47	39.2
Drinking alcohol	12	10

Abbreviations: NCD, non-communicable disease.

**Table 2:** Gender-specific anthropometric characteristics of the study population.

Variable	Men (*N* = 38)		Women (*N* = 82)		Total (*N* = 120)		*p-*Value *
	Mean	SD	Mean	SD	Mean	SD	
Age	50.11	14.66	43.50	15.85	45.59	15.73	0.032
SBP (mmHg)	150.16	25.27	142.49	20.81	144.92	22.50	0.082
DBP (mmHg)	97.39	15.58	93.89	13.91	95.00	14.49	0.219
Weight (kg)	57.67	9.87	55.05	11.28	55.88	10.88	0.221
Height (cm)	161.39	6.08	153.04	7.23	155.68	7.89	<0.001
BMI (kg/m^2^)	22.20	3.97	23.46	4.26	23.06	4.20	0.125
% Body fat	26.05	8.49	34.79	4.92	32.02	7.45	<0.001
% Body muscle	30.38	4.93	24.93	2.32	26.65	4.21	<0.001
MUAC (cm)	27.74	3.70	28.40	3.60	28.19	3.63	0.352
WC (cm)	84.79	9.38	85.96	9.53	85.59	9.46	0.529
HC (cm)	87.63	8.96	91.32	8.79	90.15	8.98	0.036
WHR	0.97	0.04	0.94	0.05	0.95	0.05	0.002
WHtR	0.53	0.06	0.56	0.07	0.55	0.07	0.005
FMI (kg/m^2^)	6.06	2.69	8.33	2.58	7.61	2.81	<0.001
HGS-L (kg)	31.17	11.27	22.96	7.01	25.56	9.36	<0.001
HGS-R (kg)	30.05	11.26	23.67	7.07	25.69	9.08	0.002

* *p*-values for comparisons between genders by independent sample *t* tests. Abbreviations: SD, standard deviation; SBP, systolic blood pressure; DBP, diastolic blood pressure; BMI, body mass index; MUAC, mid upper arm circumference; WC, waist circumference; HC, hip circumference; WHR, waist to hip ratio; WHtR, waist to height ratio; FMI, fat mass index; HGS-L, handgrip strength left hand; HGS-R, handgrip strength right hand.

**Table 3:** Distribution based on BMI, CO, NWCO and PA by gender.

Variable	Men *n* (%)	Women *n* (%)	Total *n* (%)	*p-*Value *
BMI category ^†^				
Underweight	7 (18.4)	7 (8.5)	14 (11.7)	0.321
Normal	14 (36.8)	33 (40.2)	47 (39.2)	
Overweight	14 (36.8)	29 (35.4)	43 (35.8)	
Obese	3 (7.9)	13 (15.9)	16 (13.3)	
CO ^‡^				
By WC	12 (31.6)	59 (72)	71 (59.2)	<0.001
By WHR	37 (97.4)	80 (97.6)	117 (97.5)	0.950
By WHtR	22 (57.9)	67 (81.7)	89 (74.2)	0.006
NWCO				
By WC	1 (2.6)	16 (19.5)	17 (14.2)	0.014
By WHR	13 (34.2)	31 (37.8)	44 (36.7)	0.704
By WHtR	8 (21.1)	21 (25.6)	29 (24.2)	0.588
PA level				
Low	12 (31.6)	8 (9.8)	20 (16.7)	0.003
Moderate	5 (13.2)	29 (35.4)	34 (28.3)	
High	21 (55.3)	45 (54.9)	66 (55)	

* *p*-values for comparisons between genders by chi-square tests. ^†^ BMI category: underweight, BMI < 18.5 kg/m^2^; normal, 18.5–22.9 kg/m^2^; overweight, 23–27.5 kg/m^2^; obese, >27.5 kg/m^2^. ^‡^ Central obesity: by WC, men ≥ 90 cm, women ≥ 80 cm; by WHR, men ≥ 0.9, women ≥ 0.85; by WHtR, >0.5. Abbreviations: BMI, body mass index; WC, waist circumference; WHR, waist to hip ratio; WHtR, waist to height ratio; CO, central obesity; NWCO, normal weight central obesity; PA, physical activity.

**Table 4:** Comparison of mean food intake by food groups of the study population with the national recommendations.

Food Group	Mean Intake (SD) (Portion/Day)	National Recommendation [23] (Portion/Day)	% Meets National Recommendation
Cereals and equivalents	10.90 (2.36)	6–11	100
Vegetables	1.38 (1.07)	3–5	8.3
Fruits	0.1 (0.35)	2–3	1.7
Fruits and vegetables	1.48 (1.11)	≥5	0.8
Meat, fish, eggs and/or pulses	2.76 (1.68)	3–4	44.2
Milk or milk products	0.52 (0.65)	1–2	43.3
Added sugar	3.08 (2.19)	Sparingly	13.3

Abbreviation: SD, standard deviation.

**Table 5:** Energy, macronutrient and water consumption of the study sample.

Energy and Nutrient Intake	Male (*N* = 38)		Female (*N* = 82)		Total (*N* = 120)				*p* *
	Mean	SD	Mean	SD	Min	Max	Mean	SD	
Energy (kcal/day)	1384.0	418.55	1415.56	396.49	677.40	2943.20	1430.18	387.63	0.555
Energy (kcal/kg per day)	26.11	7.78	26.13	8.36	11.55	47.88	26.14	8.66	0.986
Protein (g/day)	37.93	16.58	38.21	15.99	10.70	91.30	38.35	15.82	0.896
Protein (g/kg per day)	0.03	0.006	0.03	0.007	0.01	0.05	0.03	0.008	0.846
Fat (g/day)	32.39	21.87	29.75	19.68	1.30	100.10	28.52	18.59	0.318
Carbohydrate (g/day)	232.68	70.29	250.67	65.29	135.80	507.00	259.0	61.5	0.039
Energy from protein (%)	10.79	2.53	10.62	2.53	5.00	18.00	10.54	2.54	0.613
Energy from fat (%)	19.53	9.3	17.2	8.35	2.00	40.00	16.12	7.7	0.037
Energy from carbohydrates (%)	69.68	9.81	72.18	9.41	50.00	91.00	73.34	9.05	0.047

* Significant differences between the sexes were assessed by independent sample t-test at *p* < 0.05.

## References

[1] Weerahewa J.W.C., Babu S., Atapattu N. (2018). Food Policies and Nutrition Transition in Sri Lanka: Historical Trends, Political Regimes, and Options for Interventions.

[2] Katulanda P.C.G., Mahesh J.G., Sheriff R., Seneviratne R.D.A., Wijeratne S., Wijesuriya M., McCarthy M.I., Adler A.I., Matthews D.R. (2008). Prevalence and projections of diabetes and pre-diabetes in adults in Sri Lanka—Sri Lanka diabetes, cardiovascular study (SLDCS). Diabet. Med..

[3] Katulanda P.R.P., Jayawardena R., Sheriff R., Matthews D. (2012). Metabolic syndrome among Sri Lankan adults: Prevalence, patterns and correlates. Diabetol. Metab. Syndr..

[4] Rajapakse S., Shivanthan M.C., Selvarajah M. (2016). Chronic kidney disease of unknown etiology in Sri Lanka. Int. J. Occup. Environ. Health.

[5] Jayasumana C., Orantes C., Herrera R., Almaguer M., Lopez L., Silva L.C., Ordunez P., Siribaddana S., Gunatilake S., De Broe M.E. (2017). Chronic interstitial nephritis in agricultural communities: A worldwide epidemic with social, occupational and environmental determinants. Nephrol. Dial. Transplant. Off. Publ. Eur. Dial. Transpl. Assoc.-Eur. Ren. Assoc..

[6] Jayatilake N., Mendis S., Maheepala P., Mehta F.R. (2013). Chronic kidney disease of uncertain aetiology: Prevalence and causative factors in a developing country. BMC Nephrol..

[7] FAO (1990). Conducting Small Scale Nutrition Surveys: A Field Manual.

[8] Katulanda P., Jayawardena M.A., Sheriff M.H., Constantine G.R., Matthews D.R. (2010). Prevalence of overweight and obesity in Sri Lankan adults. Obes. Rev. Off. J. Int. Assoc. Study Obes..

[9] Anne S., Paula B. (2006). Household Dietary Diversity Score (HDDS) for Measurement of Household Food Access: Indicator Guide.

[10] IPAQ (2005). Guidelines for Data Processing and Analysis of the International Physical Activity Questionnaire (IPAQ). http://www.ipaq.ki.se/scoring.pdf.

[11] WHO (2008). Waist Circumference and Waist–Hip Ratio: Report of a WHO Expert Consultation.

[12] WHO (1995). Physical status: The use and interpretation of anthropometry. Report of a WHO Expert Committee.

[13] Mancia G., De Backer G., Dominiczak A., Cifkova R., Fagard R., Germano G., Grassi G., Heagerty A.M., Kjeldsen S.E., Laurent S. (2007). 2007 Guidelines for the management of arterial hypertension: The Task Force for the Management of Arterial Hypertension of the European Society of Hypertension (ESH) and of the European Society of Cardiology (ESC). Eur. Heart J..

[14] Shechtman O.S.B., MacDermid J., Solomon G., Valdes K., Mount Laurel N.J., American Society of Hand Therapists (2015). Grip Strength. Clinical Assessment Recommendations.

[15] (2004). Appropriate body-mass index for Asian populations and its implications for policy and intervention strategies. Lancet (Lond. Engl.).

[16] IDF (2006). The IDF Consensus: Worldwide Definition of the Metabolic Syndrome.

[17] Ashwell M., Gibson S. (2009). Waist to height ratio is a simple and effective obesity screening tool for cardiovascular risk factors: Analysis of data from the British National Diet and Nutrition Survey of adults aged 19–64 years. Obes. Facts.

[18] Foote J.A., Murphy S.P., Wilkens L.R., Basiotis P.P., Carlson A. (2004). Dietary variety increases the probability of nutrient adequacy among adults. J. Nutr..

[19] Mirmiran P., Azadbakht L., Azizi F. (2006). Dietary diversity within food groups: An indicator of specific nutrient adequacy in Tehranian women. J. Am. Coll. Nutr..

[20] Working Group on Infant and Young Child Feeding Indicators (2006). Developing and Validating Simple Indicators of Dietary Quality and Energy Intake of Infants and Young Children in Developing Countries: Summary of Findings from Analysis of 10 Data Sets.

[21] Kennedy G., Ballard T., Dop M. (2010). Guidelines for Measuring Household and Individual Dietary Diversity.

[22] Swindale A., Bilinsky P. (2006). Household Dietary Diversity Score (HDDS) for Measurement of Household Food Access: Indicator Guide.

[23] Nutrition Division: Ministry of Health Care and Nutrition (2011). Food Based Dietary Guidelines for Sri Lanka.

[24] Jayawardena R., Byrne N.M., Soares M.J., Katulanda P., Yadav B., Hills A.P. (2013). High dietary diversity is associated with obesity in Sri Lankan adults: An evaluation of three dietary scores. BMC Public Health.

[25] Rathnayake K.M., Madushani P., Silva K. (2012). Use of dietary diversity score as a proxy indicator of nutrient adequacy of rural elderly people in Sri Lanka. BMC Res. Notes.

[26] Jayawardena R., Byrne N.M., Soares M.J., Katulanda P., Hills A.P. (2013). Food consumption of Sri Lankan adults: An appraisal of serving characteristics. Public Health Nutr..

[27] Department of Census & Statistics-Sri Lanka. Official poverty line by district: January 2019. http://www.statistics.gov.lk/poverty/monthly_poverty/index.htm.

[28] Ranasinghe C., Gamage P., Katulanda P., Andraweera N., Thilakarathne S., Tharanga P. (2013). Relationship between Body mass index (BMI) and body fat percentage, estimated by bioelectrical impedance, in a group of Sri Lankan adults: A cross sectional study. BMC Public Health.

[29] Jin M., Du H., Zhang Y., Zhu H., Xu K., Yuan X., Pan H., Shan G. (2018). Characteristics and reference values of fat mass index and fat free mass index by bioelectrical impedance analysis in an adult population. Clin. Nutr. (Edinb. Scotl.).

[30] Liu P., Ma F., Lou H., Liu Y. (2013). The utility of fat mass index vs. body mass index and percentage of body fat in the screening of metabolic syndrome. BMC Public Health.

[31] Wang D., Li Y., Lee S.G., Wang L., Fan J., Zhang G., Wu J., Ji Y., Li S. (2011). Ethnic differences in body composition and obesity related risk factors: Study in Chinese and white males living in China. PLoS ONE.

[32] Norman K., Stobaus N., Gonzalez M.C., Schulzke J.D., Pirlich M. (2011). Hand grip strength: Outcome predictor and marker of nutritional status. Clin. Nutr. (Edinb. Scotl.).

[33] Massy-Westropp N.M., Gill T.K., Taylor A.W., Bohannon R.W., Hill C.L. (2011). Hand Grip Strength: Age and gender stratified normative data in a population-based study. BMC Res. Notes.

[34] Amaral C.A., Amaral T.L.M., Monteiro G.T.R., Vasconcellos M.T.L., Portela M.C. (2019). Hand grip strength: Reference values for adults and elderly people of Rio Branco, Acre, Brazil. PLoS ONE.

[35] Mathiowetz V., Kashman N., Volland G., Weber K., Dowe M., Rogers S. (1985). Grip and pinch strength: Normative data for adults. Arch. Phys. Med. Rehabil..

[36] Tveter A.T., Dagfinrud H., Moseng T., Holm I. (2014). Health-related physical fitness measures: Reference values and reference equations for use in clinical practice. Arch. Phys. Med. Rehabil..

[37] Werle S., Goldhahn J., Drerup S., Simmen B.R., Sprott H., Herren D.B. (2009). Age- and Gender-Specific Normative Data of Grip and Pinch Strength in a Healthy Adult Swiss Population. J. Hand Surg. (Eur. Vol.).

[38] Mohammadian M., Choobineh A., Haghdoost A., Hasheminejad N. (2014). Normative data of grip and pinch strengths in healthy adults of Iranian population. Iran. J. Public Health.

[39] Tsang R.C.C. (2005). Reference Values for 6-Minute Walk Test and Hand-Grip Strength in Healthy Hong Kong Chinese Adults. Hong Kong Physiother. J..

[40] Nurul Shahida M.S., Siti Zawiah M.D., Case K. (2015). The relationship between anthropometry and hand grip strength among elderly Malaysians. Int. J. Ind. Ergon..

[41] Adedoyin R.A., Ogundapo F.A., Mbada C.E., Adekanla B.A., Johnson O.E., Onigbinde T.A., Emechete A.A.I. (2009). Reference Values for Handgrip Strength Among Healthy Adults in Nigeria. Hong Kong Physiother. J..

[42] Bansal N. (2008). Hand Grip Strength: Normative Data for Young Adults. Indian J. Phys. Occup. Ther. Int. J..

[43] Kamarul T., Ahmad T.S., Loh W.Y. (2006). Hand grip strength in the adult Malaysian population. J. Orthop. Surg. (Hong Kong).

[44] Katulanda P., Dissanayake H.A., De Silva S.D.N., Katulanda G.W., Liyanage I.K., Constantine G.R., Sheriff R., Matthews D.R. (2018). Prevalence, patterns, and associations of dyslipidemia among Sri Lankan adults-Sri Lanka Diabetes and Cardiovascular Study in 2005-2006. J. Clin. Lipidol..

[45] De Silva A.P., De Silva S.H., Haniffa R., Liyanage I.K., Jayasinghe K.S., Katulanda P., Wijeratne C.N., Wijeratne S., Rajapakse L.C. (2015). A cross sectional survey on social, cultural and economic determinants of obesity in a low middle income setting. Int. J. Equity Health.

[46] Jayawardana N., Jayalath W., Madhujith W.M.T., Ralapanawa U., Jayasekera R.S., Alagiyawanna S., Bandara A., Kalupahana N.S. (2017). Aging and obesity are associated with undiagnosed hypertension in a cohort of males in the Central Province of Sri Lanka: A cross-sectional descriptive study. BMC Cardiovasc. Disord..

[47] Wijewardene K., Mohideen M., Mendis S., Fernando D., Kulathilaka T., Weerasekara D., Uluwitta P. (2005). Prevalence of hypertension, diabetes and obesity: Baseline findings of a population based survey in four provinces in Sri Lanka. Ceylon Med. J..

[48] Arambepola C., Allender S., Ekanayake R., Fernando D. (2008). Urban living and obesity: Is it independent of its population and lifestyle characteristics?. Trop. Med. Int. Health.

[49] Rathnayake K.M., Roopasingam T., Dibley M.J. (2014). High carbohydrate diet and physical inactivity associated with central obesity among premenopausal housewives in Sri Lanka. BMC Res. Notes.

[50] Jayawardana R., Ranasinghe P., Sheriff M.H., Matthews D.R., Katulanda P. (2013). Waist to height ratio: A better anthropometric marker of diabetes and cardio-metabolic risks in South Asian adults. Diabetes Res. Clin. Pr..

[51] Jayawardena R., Byrne N.M., Soares M.J., Katulanda P., Hills A.P. (2014). Body weight perception and weight loss practices among Sri Lankan adults. Obes. Res. Clin. Pract..

[52] Jayatissa R., Moazzem Hossain S.M., Gunawardana S., Ranbanda J.M., Gunathilaka M., De Silva P.C. (2012). Prevalence and associations of overweight among adult women in Sri Lanka: A national survey. Sri Lanka J. Diabetes Endocrinol. Metab..

[53] Amarasinghe S., Sandrasegarampillai B., Arasaratnam V. (2015). Metabolic syndrome among Jaffna Tamil community, Sri Lanka. Indian J. Endocrinol. Metab..

[54] Owolabi E.O., Ter Goon D., Adeniyi O.V. (2017). Central obesity and normal-weight central obesity among adults attending healthcare facilities in Buffalo City Metropolitan Municipality, South Africa: A cross-sectional study. J. HealthPopul. Nutr..

[55] Thaikruea L., Thammasarot J. (2016). Prevalence of normal weight central obesity among Thai healthcare providers and their association with CVD risk: A cross-sectional study. Sci. Rep..

[56] Zhang P., Wang R., Gao C., Jiang L., Lv X., Song Y., Li B. (2016). Prevalence of Central Obesity among Adults with Normal BMI and Its Association with Metabolic Diseases in Northeast China. PLoS ONE.

[57] Katulanda P., Jayawardena M.A., Sheriff M.H., Matthews D.R. (2010). The distance between the lower edge of the xiphisternum and the center of the umbilicus as an indicator of abdominal obesity and cardiovascular disease risk. Obes Facts.

[58] Kasturiratne A., Warnakulasuriya T., Pinidiyapathirage J., Kato N., Wickremasinghe R., Pathmeswaran A. (2011). P2-130 Epidemiology of hypertension in an urban Sri Lankan population. J. Epidemiol. Community Health.

[59] Katulanda P., Ranasinghe P., Jayawardena R., Constantine G.R., Rezvi Sheriff M.H., Matthews D.R. (2014). The prevalence, predictors and associations of hypertension in Sri Lanka: A cross-sectional population based national survey. Clin. Exp. Hypertens..

[60] Gamage A., Seneviratne R., Hanna F. (2016). Ps 17-23 physical inactivity as a predictor of hypertension among employees in Sri Lanka: a cross-sectional study. J. Hypertens..

[61] Rathnayake K.M., Wimalathunga M., Weech M., Jackson K.G., Lovegrove J.A. (2015). High prevalence of undernutrition and low dietary diversity in institutionalised elderly living in Sri Lanka. Public Health Nutr..

[62] Siriwardhana E.R.I., Perera P.A., Sivakanesan R., Abeysekara T., Nugegoda D.B., Weerakoon K.G. (2014). Is the staple diet eaten in Medawachchiya, Sri Lanka, a predisposing factor in the development of chronic kidney disease of unknown etiology?-A comparison based on urinary β2-microglobulin measurements. BMC Nephrol..

[63] Jayawardena R., Thennakoon S., Byrne N., Soares M., Katulanda P., Hills A. (2014). Energy and nutrient intakes among Sri Lankan adults. Int. Arch. Med..

[64] (2005). Human energy requirements: Report of a joint FAO/ WHO/UNU Expert Consultation. Food Nutr. Bull..

[65] (2003). Diet, nutrition and the prevention of chronic diseases. World Health Organ. Tech. Rep. Ser..

